# Food allergen thresholds: CM-allergic infants managed with hypoallergenic formulas are a more sensitive population

**DOI:** 10.1186/2045-7022-3-S3-P149

**Published:** 2013-07-25

**Authors:** C Cordle, LW Williams, G Duska-McEwen, MJ Farrow

**Affiliations:** 1 Pediatric Product Development, Abbott Nutrition, Columbus, OH, USA; 2 Abbott Nutrition, Columbus, OH, USA

## Background

Understanding the relationship of food allergen dose to clinical symptoms is important for managing food-allergic patients, the food industry, and regulatory bodies as all strive to insure food safety. Food allergen reactivity thresholds are highly variable depending on the food and the food-allergic patient population. Existing studies describe reaction thresholds for major food allergens that are useful in managing individual patients. Reaction thresholds are not distributed in a statistically “normal” fashion; some infants with cow milk allergy show sensitivity to extremely low doses. Data describing these unique patients is presented.

## Methods

A review of cow milk allergy (CMA) threshold data was conducted to identify reactions associated with specific milk intake events. Calculations were performed to identify Lowest Observed Adverse Effect Levels (LOAEL) using reported food intake amounts and allergen content (usually ELISA-based). For reports of reactions to clinically hypoallergenic formula based on extensively hydrolyzed proteins (HF), intake amounts were generally available but accurate antigen content data was not. Product matrix-corrected inhibition ELISA methods were developed and validated with model products to measure bovine casein and whey antigens in HF. The methods were used to quantify casein and whey antigen content of HF in the reports. Antigen doses causing reactions were then calculated based on reported ingested volumes. Reaction thresholds for HF-managed infants were compared to the larger data base.

## Results

14 reports of CMA reactions to intact milk protein challenges show an average LOAEL of 83.7mg milk protein (range = 0.36-280mg). 2 reports of CMA reactions from contaminated products show triggering doses of 0.71 and 16.2mg milk protein. ELISA methods to quantify bovine casein and whey were developed (accuracy + 5-25%, precision 5-20%, sensitivity < 10-25ng/mL) and used to quantify casein and whey in HF. These data were used to calculate antigen doses in infants reacting to HF. In contrast to the data on intact milk challenge, 5 reports of reactions in CMA children caused by extensively hydrolyzed HF show an average LOAEL of 6.95 micrograms milk protein (range = 1.23 – 25mg).

## Conclusion

Some milk-allergic infants managed with HF represent a “sensitive population”. Reaction threshold data from these patients suggest a milk antigen LOAEL that is approximately 10,000 X lower that seen in the general CMA population.

## Disclosure of interest

None declared.

